# Supplementary material for: Assessing the opportunity for selection to impact morphological traits in crosses between two Solanum species
Source: PeerJ. 2024 Aug 28;12:e17985. doi: 10.7717/peerj.17985 (PMC11365482; doi:10.7717/peerj.17985)
Supplement: Supplemental Information 1 — Mean and standard error of each measured phenotype for the number of observations of each cohort. N (number of observations), RPA (ratio of perimeter to area), RWL (ratio of width to length), AR (areal ratio). [file peerj-12-17985-s001.docx]

|  |  | P1 |  |  |  | P2 |  |  |  | F2 |  |  |  | BC1 |  |  |  | rBC2 |  |
| --- | --- | --- | --- | --- | --- | --- | --- | --- | --- | --- | --- | --- | --- | --- | --- | --- | --- | --- | --- |
| Trait | N | Mean | SE |  | N | Mean | SE |  | N | Mean | SE |  | N | Mean | SE |  | N | Mean | SE |
| Area | 23 | 6.523 | 0.548 |  | 14 | 16.480 | 1.680 |  | 13 | 9.740 | 1.031 |  | 14 | 11.625 | 0.940 |  | 21 | 11.296 | 0.801 |
| Perimeter | 23 | 10.155 | 0.460 |  | 14 | 23.214 | 1.500 |  | 13 | 13.379 | 0.882 |  | 14 | 14.190 | 0.869 |  | 21 | 17.532 | 0.761 |
| RPA | 23 | 1.622 | 0.212 |  | 14 | 1.580 | 0.342 |  | 13 | 1.420 | 0.348 |  | 14 | 1.261 | 0.192 |  | 21 | 1.632 | 0.242 |
| Width | 23 | 2.720 | 0.233 |  | 14 | 3.604 | 0.602 |  | 13 | 2.918 | 0.406 |  | 14 | 3.221 | 0.422 |  | 21 | 3.058 | 0.324 |
| Length | 23 | 3.206 | 0.292 |  | 14 | 7.625 | 0.763 |  | 13 | 4.901 | 0.694 |  | 14 | 5.022 | 0.440 |  | 21 | 5.825 | 0.388 |
| RWL | 23 | 0.855 | 0.117 |  | 14 | 0.473 | 0.175 |  | 13 | 0.606 | 0.167 |  | 14 | 0.639 | 0.156 |  | 21 | 0.526 | 0.109 |
| AR | 23 | 1.038 | 0.208 |  | 14 | 1.086 | 0.402 |  | 13 | 0.948 | 0.323 |  | 14 | 1.066 | 0.370 |  | 21 | 0.993 | 0.251 |
| Seed Mass | 15 | 0.459 | 0.024 |  | 15 | 3.760 | 0.195 |  | 15 | 2.319 | 0.519 |  | 15 | 1.556 | 0.113 |  | 13 | 2.782 | 0.211 |
